# The impact of the HEART risk score in the early assessment of patients with acute chest pain: design of a stepped wedge, cluster randomised trial

**DOI:** 10.1186/1471-2261-13-77

**Published:** 2013-09-26

**Authors:** Judith M Poldervaart, Johannes B Reitsma, Hendrik Koffijberg, Barbra E Backus, A Jacob Six, Pieter A Doevendans, Arno W Hoes

**Affiliations:** 1 Julius Center for Health Sciences and Primary care, University Medical Center, Stratenum 6.131, PO box 85500, 3508AB, Utrecht, the Netherlands; 2 Department of Cardiology, Zuwe Hofpoort Hospital, Woerden, the Netherlands; 3 Department of Cardiology, University Medical Center Utrecht, Utrecht, the Netherlands

**Keywords:** HEART score, Chest pain, Clinical prediction rule, Risk score implementation, Impact, Stepped wedge design, Cluster randomised trial

## Abstract

**Background:**

Chest pain remains a diagnostic challenge: physicians do not want to miss an acute coronary syndrome (ACS), but, they also wish to avoid unnecessary additional diagnostic procedures. In approximately 75% of the patients presenting with chest pain at the emergency department (ED) there is no underlying cardiac cause. Therefore, diagnostic strategies focus on identifying patients in whom an ACS can be safely ruled out based on findings from history, physical examination and early cardiac marker measurement. The HEART score, a clinical prediction rule, was developed to provide the clinician with a simple, early and reliable predictor of cardiac risk. We set out to quantify the impact of the use of the HEART score in daily practice on patient outcomes and costs.

**Methods/Design:**

We designed a prospective, multi-centre, stepped wedge, cluster randomised trial. Our aim is to include a total of 6600 unselected chest pain patients presenting at the ED in 10 Dutch hospitals during an 11-month period. All clusters (i.e. hospitals) start with a period of ‘usual care’ and are randomised in their timing when to switch to ‘intervention care’. The latter involves the calculation of the HEART score in each patient to guide clinical decision; notably reassurance and discharge of patients with low scores and intensive monitoring and early intervention in patients with high HEART scores. Primary outcome is occurrence of major adverse cardiac events (MACE), including acute myocardial infarction, revascularisation or death within 6 weeks after presentation. Secondary outcomes include occurrence of MACE in low-risk patients, quality of life, use of health care resources and costs.

**Discussion:**

Stepped wedge designs are increasingly used to evaluate the real-life effectiveness of non-pharmacological interventions because of the following potential advantages: (a) each hospital has both a usual care and an intervention period, therefore, outcomes can be compared within and across hospitals; (b) each hospital will have an intervention period which enhances participation in case of a promising intervention; (c) all hospitals generate data about potential implementation problems. This large impact trial will generate evidence whether the anticipated benefits (in terms of safety and cost-effectiveness) of using the HEART score will indeed be achieved in real-life clinical practice.

**Trial registration:**

ClinicalTrials.gov 80-82310-97-12154.

## Background

Patients presenting with chest pain at the emergency department (ED) pose a diagnostic challenge. Chest pain can be a symptom of an acute coronary syndrome (ACS), i.e. acute myocardial infarction (AMI) or unstable angina, which is the case in approximately 20% of the patients and requires prompt treatment. In the remaining 80%, chest pain is caused by many other, usually not life-threatening, conditions [1]. Unfortunately, decision-making in chest pain patients is hampered by limited predictive power of patient characteristics, including signs, symptoms and additional tests [1-3]. Therefore, physicians face the challenge of not wanting to miss an ACS on the one hand, while avoiding too many unnecessary diagnostic procedures that can be time-consuming and patient burdening on the other hand. Currently, the fear of missing a relevant cardiac condition makes physicians cautious and, to be on the safe side, a large proportion of patients are kept in the hospital from several hours to days for monitoring or additional testing.

Diagnostic strategies in patients with chest pain therefore focus on identifying patients in whom ACS can be safely ruled out based on readily available clinical findings from history, physical examination and early marker measurement of cardiac damage. Recent guidelines suggest the use of well-developed and validated risk scores to stratify patients in the emergency room [4-6]. Several prognostic risk scores have been developed for patients diagnosed *with ACS*, such as the TIMI (Thrombolysis in Myocardial Infarction) risk score and the GRACE (Global Registry of Acute Cardiac Events) risk score [7-9]. However, scores that *identify ACS* in patients suspected of ACS in the emergency setting and predict short-term mortality or coronary intervention are not available. The HEART score has been specifically developed for risk stratification in all patients with chest pain presenting at the ED.

The HEART score incorporates all five important elements of clinical judgement in chest pain patients: History, ECG (electrocardiogram), Age, Risk factors and Troponin (see Table 1). Similar to the Apgar score, applied worldwide to assess the need for intensive care in new-borns [10], each of the five elements is appreciated with 0, 1 or 2 points. The sum of all five elements results in a score between 0–10, which can easily be calculated.

**Table 1:** Elements to calculate HEART score for chest pain patients at the emergency department

**History**	Highly suspicious	2
	Moderately suspicious	1
	Slightly or non-suspicious	0
**ECG**	Significant ST-depression	2
	Nonspecific repolarization disturbance	1
	Normal	0
**Age**	≥ 65 years	2
	>45 – <65 years	1
	≤ 45 years	0
**Risk factors**	≥ 3 risk factors*, *or* history of atherosclerotic disease^	2
	1 or 2 risk factors	1
	No risk factors known	0
**Troponin**	≥ 3x normal limit	2
	>1 - <3x normal limit	1
	≤ normal limit	0
**Total**		
**Range: 0-10**		

*Risk factors include: currently treated diabetes mellitus, current or recent smoker, diagnosed and/or treated hypertension, diagnosed hypercholesterolemia, family history of coronary artery disease (CAD) , obesity (body mass index (BMI) >30).

^History of atherosclerotic disease include: coronary revascularization, myocardial infarction, stroke, or peripheral arterial disease, irrespective of the risk factors for CAD.

The HEART score has been externally validated in various patient populations with a total of 6174 patients and its predictive effectiveness has been demonstrated [11-14]. Table 2 depicts an overview of these validation studies. In the Dutch multicenter validation study, major adverse cardiac events (MACE) occurred in 1.7% (95% CI 1.18-2.22) of all patients with a HEART score of 3 or lower. This is comparable with the around 2% incidence of ACS among discharged chest pain patients reported in the literature [15,16]. Importantly, none of the patients in the low-risk HEART category experienced unexpected sudden cardiac death in our validation studies. MACE occurred in 16.6% of all patients with intermediate HEART scores (4–6), and in 50.1% of all patients with high HEART scores (7–10). Similar results were observed in relevant patient subgroups, such as women, elderly or diabetics [12]. Notwithstanding these promising validation results, the impact of the use of the HEART score in daily clinical practice remains to be established. The HEART score provides the physician with a formal risk score and a recommendation whether a chest pain patient should be admitted or not. A safe and early discharge could potentially result in a significant reduction of patient burden, hospital admissions and health care costs.

**Table 2:** Summary of results of previous validation studies of the HEART score

				**Total**
**Number of patients**	N = 880 [12]	N = 2388 [13]	N = 2906 [14]	N = 6174
**Design**	Retrospective	Prospective	Prospective	
**Countries**	The Netherlands	The Netherlands	9 countries in the Asia-Pacific region	
**Participating hospitals**	4	10	14	
**Inclusion period**	Jan ‘06 – Mar ‘06	Oct ‘08 –Nov ‘09	Nov ‘07 – Dec ‘10	
**Type of patients**	Patients presenting with chest pain at the ED	Patients presenting with chest pain at the ED	Patients presenting with chest pain at the ED	
**MACE definition**	AMI, revascularisation, all cause death	AMI, revascularisation, stenosis managed conservatively, all cause death	AMI, revascularisation, death unless clearly non-cardiac	
**Duration of follow-up**	6 weeks	6 weeks	4 weeks	
**Cumulative incidence of MACE stratified by HEART scores**				
**0-3**	0.99%	1.7%	1.7%	1.6% (95%-CI 1.05-2.15)
**4-6**	11.6%	16.6%	14.3%	12.5% (95%-CI 11.34-13.66)
**7-10**	65.2%	50.1%	50.0%	49.4% (95%-CI 46.37- 52.43)

ED: emergency department.

AMI: acute myocardial infarction.

MACE: major adverse cardiac event.

95%-CI: 95%- confidence intervals.

Therefore, we designed the HEART Impact study to investigate whether the use of the HEART score in the management of chest pain patients indeed leads to these positive health effects, while not causing an increase in the occurrence of MACE.

## Methods/Design

### Study design: stepped wedge randomised trial

**Figure 1:**
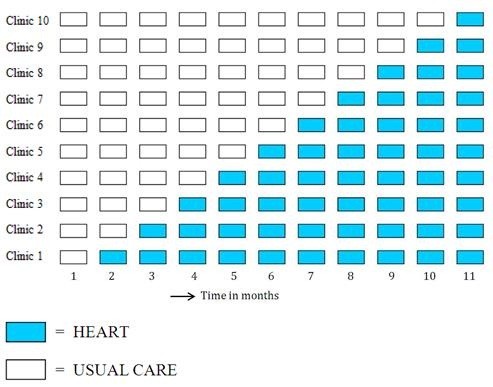
The Stepped Wedge Design for the HEART Impact study.

**Figure 2:**
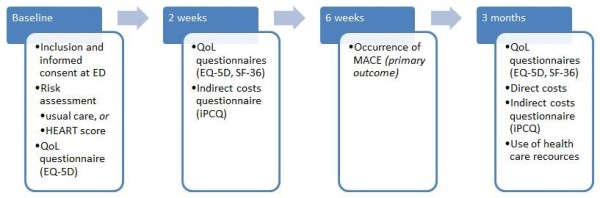
**Flow of study and data collection in usual care period and in HEART period.** ED: emergency department. QoL:quality of life. EQ-5D: EuroQol Five-Dimensional. SF-36: short-form 36. iPCQ: productivity cost questionnaire. MACE: major adverse cardiac events.

We will use a prospective, stepped wedge cluster randomised trial [17,18]. Our aim is to include 6600 unselected chest pain patients from 10 hospitals in the Netherlands during an 11-month period. Key study design features are shown in Figures 1 and 2. In a stepped wedge design, there is no randomisation at patient level, but hospitals will be randomised with respect to the timing at which they introduce the HEART score. See Figure 1. During the first month, all chest pain patients presenting to the ten hospitals will receive usual care. Then, during a 10-month period, each month one randomly allocated hospital will start to apply the HEART score (HEART period) and continue to do so until the end of the study. During the last month of the inclusion period all 10 hospitals will be using the HEART score.

### ‘Usual care’ and ‘HEART’ period

Usual care is defined as ‘daily practice of the cardiologist or attending emergency doctor to diagnose a patient with chest pain’. In this period, attending doctors assess the risk based on their clinical skills, previous experiences, gut feeling and various other criteria (for example, described in European Society of Cardiology Guidelines [6]), without using the HEART score. No attempt was made to explicitly standardise usual care across all hospitals. The assessment will typically include: gender, age, medical (cardiac) history, symptoms, risk factors, and current drug use, physical examination with special attention for the heart and lungs, blood pressure, heart rate, blood tests, ECG, and any other diagnostic procedures the physician considers necessary. The standard blood tests include measurements of troponin, glucose levels, creatinin levels (with a calculated estimated glomerular filtration rate (eGFR) according to MDRD (modification of diet in renal disease)), haemoglobin, and any other blood test required. A standard 12-lead ECG is recorded by a trained employee of the ED and classified by a single cardiologist according to the Minnesota coding criteria. All investigations take place in the ED.

During the HEART period, the HEART score will be formally determined in all patients. Decision-making about whether to admit a patient, any further testing or treatment decisions will be carried out similarly to usual care, with the exception of the availability of the HEART score in each individual patient and the recommendations linked to that score. This is also known as “directive use” of a prediction rule, as opposed to “assistive use” where only the predicted risk is given to the physician [19]. The recommendation for patients with a HEART score of 3 or lower will be reassurance and discharge. In those low-risk patients who are discharged, a second troponin will be performed at home to identify any missed ACS. A similar approach of home visits performed by ambulatory lab services was successfully applied in our earlier study in suspected ACS in primary care [20]. Obviously, in accordance with daily practice, the attending physician may decide to overrule the recommendation corresponding to a low HEART score and admit a patient. In such a case, information about the reasoning for this escape will be collected. Patients with a HEART score in the intermediate range (4–6), will generally be admitted to the hospital for further observation and investigation. The high-risk group (7–10) will typically receive prompt (invasive) treatments.

### Study population and recruitment

All patients above 18 years presenting with chest pain to the (cardiac) ED of 10 participating hospitals are eligible. Only patients presenting with evident ST-segment elevation myocardial infarction (STEMI) will be excluded, since there is no diagnostic dilemma in these patients. Typically, such patients are directly taken to the intervention room. In both study periods, information about the study procedure will be provided by the treating physician and written consent for the use of data and follow-up is obtained at the first appropriate moment after presentation at the ED. In the HEART period, no consent from the patient is needed for the use of the HEART score, for several reasons. First, the number of additional procedures for patients is minimal since the HEART score consists of elements that are collected routinely. Furthermore, the HEART score is proven to be safe, and is a decision support tool rather than a real intervention, with the possibility for physicians to override the recommendations provided by the rule. The study was approved by the Institutional Review Board, and subsequently by the Boards of the participating hospitals.

### Outcome measures

The HEART impact trial aims to measure both the intended positive changes as well as any unintended negative effects associated with the use of the HEART score. Patient outcomes, use of health care resources and costs will be determined in both periods.

#### Primary outcome: occurrence of MACE

The primary outcome is the 6-week occurrence of major adverse cardiac events (MACE), consisting of the following events: acute myocardial infarction (AMI), Percutaneous Coronary Intervention (PCI), Coronary Artery Bypass Grafting (CABG) surgery, or death due to any cause. To identify MACE after discharge, a phone-call will be made to all patients at home after 3 months. Any information that could indicate to possible endpoints will be further investigated through hospital charts, hospital discharge letters and information obtained from the patient’s general practitioner (GP). In addition, the Central Bureau for Statistics (CBS) will be consulted for information on vital status as the cause of death of participants. All cases with possible endpoints are reviewed by two independent adjudicators for endpoint classification. This adjudication committee will evaluate all relevant information to decide, using ESC guidelines, whether MACE occurred. In case of disagreement between two adjudicators, the case is discussed in a plenary adjudication committee meeting until consensus is reached.

Secondary outcomes include the following:

•The occurrence of MACE in the specific subgroup of patients with a low HEART score.

•Use of health care resources. The number of hospital admissions/discharges, duration of hospital stay, duration of stay on the ED, number of readmissions and GP visits after discharge will be collected.

•Health-related quality of life. This will be determined in a subset of approximately 1000 patients, in both time periods in five of the participating hospitals. Data on health-related quality of life are collected at baseline (at ED) using the EuroQol Five-Dimensional (EQ-5D) questionnaire, and a 2-week and at 3-month follow-up using the short form-36 (SF-36) and the EQ-5D questionnaires. SF-36 is a short-form health survey with only 36 questions. For this study, we will only use the 11 questions addressing Health. EQ-5D comprises the following 5 dimensions: mobility, self-care, usual activities, pain/discomfort and anxiety/depression. Furthermore, the EQ VAS (visual analogue scale) records the respondent’s self-rated health on a vertical, visual analogue scale where the endpoints are labelled ‘Best imaginable health state’ and ‘Worst imaginable health state’. Higher scores are associated with a better health-related quality of life.

•Direct and indirect costs. These will be determined in a subset of approximately 1000 patients, in both time periods in five of the participating hospitals*.* Actual medical costs using a health-care provider’s perspective are obtained in both the time periods. Medical resource use is extracted from the electronic hospital patient files. Unit cost prices will be determined in two participating hospitals, one academic and one peripheral hospital, using micro-costing if possible and top down costing otherwise. The iPCQ (Productivity Cost Questionnaire) will be used to collect quantitative data on the relation between illness, treatment and work performance. The iPCQ is divided into 3 modules: (1) reduced productivity at paid work due to work absenteeism, (2) reduced productivity at paid work without absence from work and (3) unpaid labour production.

•An additional sample of blood will be collected from patients of two participating hospitals during the emergency visit for future biomarkers investigation (Biobanking).

### Statistical analyses

The 6-week cumulative incidence of MACE in both the intervention and usual care period will be analysed at the patient level using a generalised linear mixed model (GLMM) [21]. Risk differences with corresponding 95% CIs will be estimated from this model. No baseline differences in prognostic factors between patients included in both periods are expected, but in case these do occur, covariates will be added to the GLMM model to adjust for these baseline differences. Differences in health-related quality of life at baseline, 2 weeks and at 3 months will also be assessed, separately for the different questionnaires. Costs per patient will be calculated according to Dutch guidelines for pharmaco-economic analyses [22], and costs of drugs prescribed will be based on Dutch formulary cost-prices.

### Sample size

The aim of the HEART study is to evaluate whether the use of the HEART score streamlines the further management of chest pain patients, in particular whether it can identify low-risk patients who can be discharged sooner than usual. However, these benefits become only relevant if the use of the HEART score does not lead to an increase in adverse cardiac events. Our sample size calculation is therefore based on demonstrating that proportion of patients with MACE is not inferior to the proportion observed with usual care. The proportion MACE expected during usual care is 17%. The non-inferiority margin is based on clinical judgement and available literature as 3%, thus accepting an upper limit of the 95% confidence interval (CI) during the intervention period of 20%. With 10 hospitals, inclusion of 60 patients per hospital per month, a between-hospital variation in incidence of 16 to 18%, a one-sided alpha of 5% and a power of 80%, 6600 patients with chest pain should be included in total. Taken into account our inclusion rates of previous validation studies and with special attention and encouragement for inclusion, we expect a realistic inclusion rate of 60 patients per hospital per month.

### Cost-effectiveness analysis

A cost-effectiveness analysis will be performed from a societal perspective, for a 3-month and a life-time time horizon. The 3-month time horizon corresponds to the actual follow-up period and will consider the observed differences in costs and quality of life. A GLMM will be used to assess cost-effectiveness, accounting for the randomisation of clusters instead of patients. Uncertainty will be addressed through the GLMM model which will be extended with cluster and patient-level covariates if baseline characteristics are imbalanced. The life-time horizon will be applied to account for long-term costs and effects of the observed MACE. Here, the observed risks of MACE, as well as the direct treatment cost and productivity losses estimated using the friction cost approach, will serve as input for a Markov decision-analytic model [23]. If necessary, additional evidence on long-term costs and effects of adverse events will be obtained from the literature. Monte Carlo simulation will be applied to simulate the course of hypothetical patients through the model, and to estimate the number of quality-adjusted life years and costs of both strategies. Costs will be discounted with 4% per annum, and effects with 1.5% per annum, according to Dutch guidelines. The incremental cost-effectiveness ratio and the net monetary benefit (for various willingness to pay thresholds) will be estimated for the HEART score compared with usual care. Uncertainty will be assessed with probabilistic sensitivity analysis [24], and results will be presented in incremental cost-effectiveness planes and cost-effectiveness acceptability curves.

### Regulation statement

This study will be conducted according to the principles of the current version of the declaration of Helsinki and in accordance with the Dutch law on Medical Research Involving Human Subjects Act (WMO).

### Ethics committee approval

The study was approved by the Institutional Review Board (medical ethical committee of the University Medical Center Utrecht, the Netherlands), and subsequently by the Boards of the participating hospitals.

## Discussion

Clinical prediction rules, like any other health care intervention, need proper evaluation before wide-spread use in clinical practice. Two key steps in this evaluation include external validation and impact assessment. External validation studies can reveal several problematic issues associated with the use of a clinical prediction rule [25-27]. Firstly, the rule has been developed on a dataset that was too small in relation to the number of variables that have been considered. This increases the risk that particularities of the dataset will be modeled rather than robust relationships. The consequence is that the performance of the model will decrease when applied to new patients (external validation). This is known as over-fitting. Secondly, the rule has been derived in a population which does not match the population where clinicians would like to use the rule. Here, your prediction model may be developed statistically sound, but applied to a new population the performance may decrease meaningfully. However, even after proper development and good performance in validation studies, often clinical prediction rules are hardly or incorrectly used in daily practice, because of difficulties in application or because physicians are not convinced of its usefulness in clinical practice. This is especially the case when the outcome used in the rule has no direct relevance for clinical practice. For all these reasons, it is of vital importance to study the impact of a clinical prediction rule when applied in real-life practice. Increasingly, stepped wedge designs are applied to measure the impact of clinical decision rules in clinical practice [28]. The stepped wedge design combines elements of both the cluster randomised trial and the before-after design (see Figure 1). The stepped wedge design has several features that make this design attractive for such impact studies. These characteristics are outlined in Table 3. We chose the stepped wedge design as an informative, efficient and valid design to examine whether expected improvements in patient outcomes, use of health care resources, and costs can be achieved when implementing a health care intervention on a large scale.

**Table 3:** Overview of key characteristics of the stepped wedge design

(i) →	Stepped wedge design has features of cluster randomisation, i.e. during a specific time period only type of intervention (usual care or HEART score) is administered
a. →	This reduces the risk of contamination
b. →	The effect of clustering needs to be taken into account in the statistical analysis
(ii) →	Stepped wedge design has features of a one direction cross-over trial, i.e. each hospital contributes data from both usual care and HEART score in a fixed order.
a. →	Allows for comparison of results within hospitals which may be less confounded by differences in case mix than between hospitals
b. →	The fixed order from usual care to HEART score further reduces the risk of contamination as the HEART score is relatively simple to calculate.
c. →	Due to the cross-over, each hospital will provide data about the (problems in) implementation of the HEART score
(iii) →	Switch from usual care to HEART score in hospitals is evenly and randomly distributed over calendar time
a. →	This reduces the impact of potential changes over time in other factors than the intervention
b. →	It facilitates the close monitoring and logistic of all activities surrounding the switch
(iv) →	Gradual implementation of new strategy is carried out, thereby providing data about the process itself.

## Conclusion

It is of importance to generate valid evidence that the use of the HEART score compared to usual care is safe and leads to fewer admissions and diagnostic procedures in real-life clinical practice. Using the stepped wedge design, we can also monitor the process of implementation of a clinical support tool at the ED across hospitals that vary in size and population. Patient inclusion has started July 1st of 2013.

## Competing interests

The authors declare that they have no competing interests.

## Authors’ contributions

AW, JB and AJ designed the study. JM, JB, H, and AW drafted the manuscript. All authors read and approved the final manuscript.

## Pre-publication history

The pre-publication history for this paper can be accessed here:

http://www.biomedcentral.com/1471-2261/13/77/prepub
